# Trends in Adult and Elderly Vaccination: Focus on Vaccination Practices in Tunisia and Morocco

**DOI:** 10.3389/fpubh.2022.903376

**Published:** 2022-07-01

**Authors:** Redouane Abouqal, Maher Beji, Mohamed Chakroun, Kamal Marhoum El Filali, Jihane Rammaoui, Hela Zaghden

**Affiliations:** ^1^Laboratory of Biostatistics, Clinical and Epidemiological Research, Faculty of Medicine and Pharmacy, Mohammed V University in Rabat, Rabat, Morocco; ^2^Acute Medical Unit, Ibn Sina University Hospital, Rabat, Morocco; ^3^Department of Internal Medicine, Military Hospital Bizerte, Bizerte, Tunisia; ^4^Faculty of Medicine of Tunis, University El Manar, Tunis, Tunisia; ^5^Tunisian Society of Tropical Medicine and Travel, Tunis, Tunisia; ^6^Infectious Diseases Department, University Hospital, Monastir, Tunisia; ^7^Infectious Diseases Department, Medical School of Casablanca, Casablanca, Morocco; ^8^Pfizer Inc., Casablanca, Morocco; ^9^Pfizer Inc., Tunis, Tunisia

**Keywords:** adult vaccination, Tunisia, Morocco, vaccine-preventable diseases, vaccination practices

## Abstract

Vaccine preventable diseases (VPDs) are a prevailing concern among the adult population, despite availability of vaccines. Unlike pediatric vaccination programs, adult vaccination programs lack the required reach, initiative, and awareness. Clinical studies and real-world data have proven that vaccines effectively reduce the disease burden of VPDs and increase life expectancy. In Tunisia and Morocco, the national immunization program (NIP) focuses more on pediatric vaccination and have limited vaccination programs for adults. However, some vaccination campaigns targeting adults are organized. For example, influenza vaccination campaigns prioritizing at risk adults which includes healthcare professionals, elderly, and patients with comorbidities. Women of childbearing age who have never been vaccinated or whose information is uncertain are recommended to receive tetanus vaccination. Tunisia NIP recommends rubella vaccine mainly for women of childbearing age, while in Morocco, national vaccination campaigns were organized for girls and women (up to 24 years of age) to eliminate rubella. Further, travelers from both countries are recommended to follow all requirements and recommendations in the travel destination. The objective of this manuscript is to provide an overview of the global disease burden of common VPDs including (but not limited to) meningococcal diseases, pneumococcal diseases, hepatitis, and influenza. The review also provides an overview of clinical data and guidelines/recommendations on adult vaccination practices, with special focus on Tunisia and Morocco. Some European and North American countries have concrete recommendations and strategies for adult vaccination to keep the VPDs in check. In Morocco and Tunisia, although, there are sporadic adult vaccination initiatives, the efforts still need upscaling and endorsements to boost vaccination awareness and uptake. There is a need to strengthen strategies in both countries to understand the disease burden and spread awareness. Additional studies are needed to generate economic evidence to support cost-effectiveness of vaccines. Integration of private and public healthcare systems may further improve vaccination uptake in adults.

## Introduction

Vaccines are an important public health tool used to prevent the spread of contagious diseases and saving lives from preventable diseases (1–3). They have improved the general life expectancy of the global population by combating mortality and morbidity associated with infectious diseases (4).

Most of the vaccination programs have focused on pediatric population (4, 5). Globally, several national and international collaborative initiatives have ensured in achieving a vaccination uptake rate of >90% in the pediatric population across many countries (5). However, the vaccination programs for adults worldwide are comparatively lower, despite the evidence of the increased burden of vaccine-preventable diseases (VPDs) in adults (4, 5).

The life expectancy has improved considerably rising from 64.2 years in 1990 to 72.6 years in 2019, and in many low- and middle-income countries (LMIC), the share of the population aged more than 65 years is expected to be double between 2019 and 2050 (6, 7). In this context, it is imperative to prioritize the health of the adult population and facilitate healthy aging strategies to manage adult mortality and morbidity, improve quality of life, and reduce disability in every way possible to have a positive impact on global wellbeing (7). The susceptibility to infectious diseases is more among the older adults, resulting in higher incidence with increased severity. The possible reason is the onset of immunosenescence (age-related dysregulation of the immune system) in the elderly population (>64 years) (8). Immunosenescence also leads to inadequate response to vaccinations which can be further impacted by other underlying conditions (5, 9). Hence, vaccination at an earlier stage in adulthood may provide appropriate immunity against infectious diseases in older adults (5). Immunization in adults is important to restore their weakening immunity against VPDs and grant immunity against other diseases. Another challenge among the older adults is the high risk for multi-drug resistance infections due to factors such as weak immune system or staying in old age homes. Vaccinations can be an efficient tool in adults that may help decrease disease burden and reduce the use of antibiotics (10). Further, immunization of adults may also prevent the spread of infectious diseases to children and other vulnerable populations.

Several clinical studies on vaccination in adults have proven to be effective (11, 12). Despite the clinical evidence, vaccination coverage in adults is not satisfactory (4) and the impact is further substantial in LMIC (including Tunisia and Morocco), as policies for adult vaccination are not widely established and implemented (13). The obvious lacunae in terms of guidelines/recommendations, and the establishment and/or implementation of vaccination programs need to be addressed. Further, information on the current disease burden of VPDs is important, while constructing preventive measures and prioritization (14). An understanding of the disease burden, existing guidelines/recommendations, and information on ongoing adult vaccination programs will help in leveraging the previous experience and comprehending the gaps to develop robust, effective, and successful vaccination programs in LMIC, where advocacy and implementation are in infancy. Further, emphasis on healthcare education and awareness about the importance of vaccination will also aid in increasing vaccination coverage in adults.

This manuscript aims to provide an overview of the global disease burden of VPDs including meningococcal diseases, pneumococcal diseases, hepatitis, influenza, human papillomavirus, diphtheria, pertussis, tetanus, rabies, measles, and tuberculosis (scope of the review does not cover information on coronavirus disease and associated vaccinations). Additionally, the review focuses on clinical studies related to adult vaccination and established guidelines/recommendations on vaccination practices in adults aged 18 years or above, with special focus on Tunisia and Morocco.

## The Burden of Vaccine Preventable Diseases

Routine immunization programs conducted worldwide for several decades have drastically reduced or eradicated infectious diseases among children saving millions of lives. In the current scenario, with an increase in aging population, there is an immediate need to focus on adult vaccination to reduce the burden of VPDs in adults. Invasive bacterial diseases and viral diseases are a major public health concern in adult populations worldwide and more specifically in LMIC, such as those in the Middle East and North Africa region, despite the availability of vaccinations for these diseases. The most common VPDs include meningococcal infections caused by *Neisseria meningitidis* (*N. meningitidis*), pneumococcal disease, caused by *Streptococcus pneumoniae* (*S. pneumoniae*), influenza caused by *Haemophilus influenzae (H. influenzae)* and *Myxovirus influenzae*, human papillomavirus (HPV), hepatitis A and B, tuberculosis, diphtheria, tetanus, pertussis, and rabies (15). An overview of the disease burden of the above mentioned VPDs will help strategize vaccination policies and practices to effectively control and prevent these diseases. The burden of VPDs obtained from various studies globally and in Africa, Morocco, and Tunisia has been summarized in Table 1.

**Table 1:** Global burden of invasive VPDs.

**S. No**.	**Name of the disease**	**Region**	**Prevalence**	**Morbidity/mortality**
1	Meningococcal diseases	Worldwide	1.2 million/year (15)	>10% 135,000 deaths/year (16)
		Sub-Saharan Africa	26,029/year (17)	2,080 deaths (17)
		Morocco	13.22/100,000 (18)	0.22/100,000 (18)
		Tunisia	6.80/100,000 (18)	0.08/100,000 (18)
2	Pneumococcal diseases	Worldwide	14.5 million/year (19)	500,000 deaths/year (19)
3	Measles	Worldwide	150,000 cases (20)	NA
		Africa	110,000 cases (20)	
		Morocco	Around 500 cases (20)	
		Tunisia	Around 20 cases (20)	
4	Influenza	Worldwide	NA	290,000–650,000 deaths/year (21)
		Sub- Saharan Africa		2.8–16.5 deaths/ 100,000 (21)
5	Human papillomavirus (HPV)	Worldwide	527,000 cases/year (22)	265,653/year (22)
		Morocco	2,058 cases/year (22)	1,076/year (22)
		Tunisia	NA	Around 1,000/year (23)
6	Pertussis	Worldwide	65,000–150,000 cases/year (24)	NA
		Africa	3,000–5,000 cases/year (24)	
		Morocco	Around 100 cases (25)	
		Tunisia	Around 20 cases (25)	
7	Hepatitis A	Worldwide	1.5 million/year (26)	7,134 deaths (20)
8	Hepatitis B	Worldwide	1.5 million/year (27)	800,000 deaths (27)
		Africa	2–20% (28)	
		Morocco	1.8–2.5% (29)	
		Tunisia	1.7% (30, 31)	
9	Rabies	Worldwide	NA	59,000 deaths/year (32)
		Africa		NA
		Morocco	22 cases/year (33)	NA
		Tunisia	4.5 cases/year (34)	
10	Diphtheria	Worldwide	10,000 cases/year (35)	
		Africa	5,300 cases/year (35)	
		Morocco	No reported cases (35)	
		Tunisia	No reported cases (35)	
		Africa		
		Tunisia	No reported cases (36)	
11	Tuberculosis	Worldwide	10 million (37)	1.3 million deaths (37)
		Tunisia	36/100,000 (38)	
		Morocco	98/100,000 (38)	

*NA, Not available*.

### International and Regional/Local Epidemiology of Vaccine Preventable Diseases Among Adults

#### Meningococcal Infection

The distribution of invasive meningococcal disease (IMD) varies by year, region, age group, and causative organism (39, 40). Globally, close to 1.2 million cases of IMD are reported annually with an overall case mortality rate of 5–10%, which includes significantly high contribution from adults aged ≥65 years (15). The highest IMD incidence is observed in infants (due to lack of natural immunity), but a second peak is seen in adolescents and young adults (due to increased risk factors such as social behavior) (41, 42). Based on capsular polysaccharides of *N. meningitidis*, 12 serogroups are identified and among them six serogroups (A, B, C, W, X, and Y) are responsible for nearly all infections globally (43). In Africa, until recently, most meningitis epidemics in the meningitis belt of sub-Saharan Africa were due to serogroup A. Since the successful rollout of a new serogroup A meningococcal conjugate vaccine in 2010, the incidence of serogroup A is declining, however other serogroups still cause epidemics. Currently, serogroup W is responsible for most cases (79%), followed by serogroup X (11%) (44). In South Africa, the distribution of IMD by serogroup is W (42%), B (21%), “not defined” (15%), and Y (13%) (44). In Morocco, IMD is considered endemic with estimated incidence and death rates for meningococcal meningitis as 13.24/100,000 and 0.22/100,000, respectively (18). While, Tunisia has relatively lower incidence and death rates estimated to be 6.80/100,000 and 0.08/100,000, respectively (18). Meningococcal serogroup B (MenB) is highly prevalent in most of North Africa (45). According to the most recent real-time PCR based surveillance study on invasive bacterial infections in North Africa (Tunisia, Algeria and Morocco), serogroup B meningococci (MenB) was the most prevalent of *N. meningitidis* ranging between 50 and 90%. Other serogroups detected in these countries were C and Y while serogroup W was not detected (46).Yet, considerable genetic heterogeneity exists across the region. For example, most MenB isolates in Morocco had the B:4:P1.15 phenotype (47), whereas NT:NST was the most frequent in Tunisian children (48). Additionally, MenB isolates in Tunisia were diverse, with six serotypes and eight sero-subtypes identified (18, 49).

#### Pneumococcal Infection

Pneumococcal infections affect people of all ages, but children younger than 2 years of age and adults aged 65 years and older are at higher risk. A limited number of *S. pneumoniae* serotypes are responsible for most serious pneumococcal infections in both adults and children around the world (50). Invasive pneumococcal disease (IPD) is often associated with high risk of morbidity and mortality. This risk is further enhanced in immunocompromised patients. A meta-analysis studying the incidence of IPD in adults (≥18 years) reported that the incidence in healthy controls was 10/100,000 person years as compared to a very high incidence range of 65/100,000–812/100,000 in immunocompromised patients with chronic inflammatory diseases, human immunodeficiency virus (HIV) and patients who underwent transplantation (51). Another observational study conducted in the United States reported that the incidence rate of IPD is 10.5 times higher in immunocompromised patients as compared to healthy adults in the age group of 19–64 years (52). The high incidence of IPD in immunocompromised patients highlights the importance of vaccination for this population. In Netherlands, the estimated average annual disease burden expressed using the disability-adjusted life years (DALY) for non-invasive pneumococcal disease was 37,223 DALYs/year, and IPD was 6061 DALYs/year (14). In LMIC, pneumococcal disease, caused by *S. pneumoniae* is estimated to affect 14.5 million children below 5 years of age, every year with 500,000 deaths (19). Other than young children, adults aged 65 years and older are at higher risk having an incidence of 1 per 1,000 adults per year (53). During Hajj pilgrimage, community-acquired pneumonia (CAP) was the leading cause of hospitalization (15–40% of hospital admissions), accounting for 22–31% of ICU admissions. CAP patients had 10% case-fatality rate, with higher rates in ICU patients (54). *S. pneumoniae* is the cause of pneumonia in at least 18% of cases during Hajj, with increased risk and higher fatality among individuals with comorbidities and elderly males (54). Other causative pathogens were also reported for CAP, such as *Staphylococcus aureus, H. influenzae*, and influenza A virus, but *S. pneumoniae* has a high clinical burden (54).

#### Influenza

An average of 389,000 (290,000–650,000) deaths due to respiratory illnesses were estimated to be related to influenza, globally each year during 2002–2011. Among these, 67% cases were reported in the adult population ≥65 years. As per the age-mortality patterns, the highest percentage of deaths in older individuals occurred in Europe (84%), and the lowest in sub-Saharan Africa (36%) (21, 55). In the United States, the Centers for Disease Control and Prevention (CDC) estimates ranged from 140,000 to 710,000 for flu-related hospitalizations and about 12,000 to 56,000 deaths since 2010 (56). A study estimating seasonal influenza-associated mortality suggested the highest mortality in sub-Saharan Africa was 2.8–16.5 deaths per 100,000 individuals and among older adults aged ≥75 years (57). Seasonal influenza also causes lower respiratory tract infections (LRTIs), which is the leading cause of infectious disease mortality. According to the global burden of disease study, 2017, influenza was globally responsible for 11.5% cases of LRTI in all age groups, with the highest number in the age group of 30–54 years. In Morocco, the incidence reported was 1,435/100,000 cases and hospitalizations due to LRTI was 237/100,000, while in Tunisia the incidence was 903/100,000 cases and 153/100,000 hospitalizations among all age groups (58).

#### Human Papillomavirus

HPV is the most common sexually transmissible infection in the world with considerably high prevalence in developing countries (42.2%) compared to more developed countries (22.6%) (59). Asia (45.5%) and Africa (29.6%) have the highest prevalence with serotypes 16 (9.5%) and 18 (6.2%) to be the prevalent type, irrespective of the region of study (60). There is considerable variability in HPV genotypic prevalence based on the geographical region, for example, serotypes 6 and 11 are most common in United States, but has low prevalence in Africa. The most infected age group was adolescent girls and young women <25 years of age, although a rebound in HPV infection in adults >45 years old was seen in Sub-Saharan Africa and American regions (59, 61). The global prevalence rate of HPV infection in men is similar to women (highest in men of the sub-Saharan Africa) (59, 62). The incidence of HPV is higher in men living with HIV and men who have sex with men than in heterosexual men (59).

In 2020, cervical cancer accounted for 604,127 new cases and 341,831 deaths worldwide. Almost all the cases of cervical cancer (99.7%) have been associated with HPV (22, 63). Morocco and Tunisia have high prevalence of HPV with an estimation of ~2,258 new cervical cancer cases annually in Morocco, and around 1,000 cervical cancer deaths occurring each year in Morocco and Tunisia (22, 23).

#### Hepatitis

Hepatitis A has a high rate of infection, with 1.5 million cases each year (26), but is less likely to cause chronic liver disease. The World Health Organization (WHO) estimated that in 2016, 7,134 patients died from hepatitis A worldwide, with most cases in LMIC (20). In many African countries including Tunisia, prevalence of hepatitis A has decreased with the improvement of sanitary and socioeconomic conditions; they have transitioned from high endemicity to low (64). In Morocco, 222 cases of hepatitis A were reported in 2015 (65).

According to WHO in 2019, 296 million people were reported with chronic hepatitis B infection leading to 820,000 deaths, mostly from cirrhosis and primary liver cancer (27, 29). About 2.7 million (7.4%) of the 36.7 million people living with HIV (PLHIV) are also infected with hepatitis B virus (HBV) of which 71% co-infected patients are from sub-Saharan Africa (66). In Africa, the seroprevalence of HBV infection ranges from 3 to 20% (28). Several studies reported Morocco as low endemic region with seroprevalence around 1.8%, and the males showed higher prevalence than females in all the age groups, especially among aged 30–50 years (29). In Tunisia, seroprevalence was 1.7% with significantly higher prevalence in males and in the ages >20 years (30, 31). Both Morocco and Tunisia are characterized by prevalence of genotype D (29).

### Other Vaccine Preventable Diseases

According to the global tuberculosis report of 2021, an estimated 9.9 million people were suffering from tuberculosis with an estimated 1.3 million deaths worldwide (37). Around 8% of infected people are living with HIV, with highest proportion of co-infected cases in African countries. In a 22-year study in Tunisia, annual incidence rate was 13.91/100,000 population/year with mortality rate of 0.39/100,000 population/year. Elderly population (age group >60 years) showed significantly higher incidence and mortality rates as compared to all other age groups (67). In 2020, the World Bank-reported incidence rate in Tunisia was 36/100,000 and in Morocco was 98/100,000 (38). Morocco has made significant progress in the control and management of tuberculosis during the past 30 years, but with COVID-19 pandemic, new challenges resurfaced as health service delivery was disrupted (68).

Pertussis is another VPD, which remains a concern in many regions despite vaccination (69). According to WHO, the prevalence of pertussis has decreased from 150,000 cases to 65,000 cases from 2019 to 2020 (28). Most cases of pertussis are among children <1 year old, however, adults are considered as the main reservoir of transmission of the disease to unvaccinated young children (30). There is a lack of surveillance in Africa and the burden of pertussis among adults is not well-reported, and likely underestimated (70).

Measles can be serious in all age groups. The reported cases of measles globally, as reported by WHO was ~150,000 in 2020 with a majority of cases (115,364) being reported from Africa (36). Specific sub-groups in a population are more likely to suffer from measles complications, which include adults older than 20 years of age, pregnant woman, and people with compromised immune systems, such as leukemia and HIV infection (71). In 2019, according to the World Bank data, Morocco had Africa's highest rates of immunization against measles, at 99% among children. However, with 483 measles cases reported in 2018, efforts are still needed for eliminating the disease (20). For Tunisia, in 2019, the Ministry of Health responded to a large measles outbreak in the country. A total of 909 cases with laboratory confirmation were reported. The two most affected age groups were those older than 15 years (31%) and infants between the ages of 6 and 12 months (28%). Death was reported in 30 cases ranging in age from 15 days to 41 years of age (median, 7.5 months) (72).

Rabies kills around 60,000 people every year worldwide, and the majority of the victims are in developing countries of Africa and Asia (32). Morocco reported 22 cases per year and Tunisia had 4 to 5 cases per year (33, 34). Most of the rabies cases are not even reported, so there is need for more awareness for better rabies control (32). The vaccination coverage for people exposed to risk is also low (33).

The aforementioned clinical disease burden suggests that VPDs contribute to a substantial proportion of infectious diseases globally, especially in African countries. However, the incidence of VPDs in adults is under-reported in most parts of the world, and hence the impact maybe even more than we currently foresee.

## Overview on Vaccines for Vaccine Preventable Diseases and Clinical Studies on Adult Vaccination

Clinical studies have extensively evaluated the efficacy, safety, and tolerability of vaccines in different age groups. An in-depth understanding of the effectiveness and tolerability of vaccines in adults is essential for both healthcare providers and government bodies to make informed decisions and establish efficient policies and recommendations. This section provides an overview of vaccines available for prevalent diseases in the North African region, which includes vaccines for meningococcal diseases, pneumococcal diseases, hepatitis B, *Haemophilus influenzae* type b and HPV (Table 2).

**Table 2:** Clinical studies for vaccination in adults.

**S. No**.	**Phase of study**	**Vaccine**	**Place of study**	**Study population (age and sample size)**	**Primary results**
**A. Vaccination in adults for meningococcal diseases**					
1	Phase 3 (73)	MenACWY-TT MenPS	Lebanon	56–103 years, *N* = 400	• Immunogenicity achieved with single dose of MenACWY-TT and MenPS • 93% of subjects achieved rSBA titers above the seroprotective threshold • VR rate in the MenACWY-TT group was 76.6%, 80.3%, 77.5% and 81.9% for serogroups A, C, W-135, and Y • VR rates in the MenPS group were 91.7%, 84.8%, 87.1%, and 89.1%, for serogroups A, C, W-135, and Y • Low anti-TT antibodies in this population • rSBA, VR, and GMTs lower in subjects over 65 years compared to 56–65 years
2	Phase 2b (74)	MenACWY-TT MenTT	Philippines and Saudi Arabia.	11–55 years, *N* = 500	• Protection for 72% of vaccinated subjects with single dose of MenACWY-TT at least 5 years post-vaccination • MenACWY-TT showed higher long-term immunogenicity than MenTT for serogroups A, W, and Y • rSBA titers ≥1:8 and ≥1:128 in 71.6–90.0% and 64.9–86.3% of MenACWY-TT recipients compared to 24.8–74.3% and 21.0–68.6% of MenACWY-PS recipients, for the total cohort year 5
3	Phase 3 (75)	MenACWY-TT Fluarix™	Lebanon and Philippines	18–55 years, *N* = 1,352	• Coadministration of ACWY-TT with seasonal influenza vaccine non-inferior compared to ACWY-TT in terms of rSBA GMTs to serogroups A, W-135, and Y • Post-vaccination rSBA GMT significantly higher in the coadministration group than MenPS group for serogroups A, W-135, and Y, and similar for serogroup C • rSBA titers ≥1:128 for all serogroups in 97% of subjects
4	Phase 3 (76)	MenACYW-TT MPSV4	USA, Puerto Rico	>56 years, *N* = 907	• Seroresponse for MenACYW-TT non-inferior to MPSV4 for each serogroup (58.2 vs. 42.5%; 77.1 vs. 49.7%; 62.6 vs. 44.8%, 74.4 vs. 43.4%, for serogroup A, C, W, and Y, respectively) • hSBA titers higher for all serogroups in MenACYW-TT subjects than MPSV4 (77.4–91.7% vs. 63.1–84.2%, respectively)
5	Phase 3 (77)	MenACYW-TT or MCV4-DT	USA	10–55 years, *N* = 3,344	• Seroresponse for MenACYW-TT group non-inferior to MCV4-DT (74 vs. 55%; 89 vs. 48%; 80 vs. 61%; 91 vs. 73%, for serogroup A, C, W, and Y, respectively) • MenACYW-TT and MCV4-DT had similar safety profiles
6	Phase 3 (78)	MenB bivalent rLP2086 or Hepatitis A vaccine	USA	10–25 years, *N* = 5,712	• Serious adverse events were rare, bivalent rLP2086 and HAV/saline recipients reported by 1.6 and 2.5%, respectively • 7.0 and 6.1% of subjects reported medically attended adverse events after vaccination dose 1; 5.5 and 6.1% after vaccination dose 2; and 5.3 and 5.5% after vaccination dose 3 in the bivalent rLP2086 and HAV/saline groups, respectively
7	Phase 3 (79)	MenACWY-D	Japan	2–55 years, *N* = 200	• MenACWY-D induced a robust immune response • Seroprotection rates for serogroups A, C, W, and Y were 91.2%, 80.2%, 89.1%, and 93.8%, respectively
8	Phase 4 (80)	MenACWY-D	Korea	2–55 years, *N* = 671	• 45% of subjects showed adverse events • Solicited and unsolicited adverse reactions reported in 17.4 and 37.9% of subjects, respectively • No vaccine related serious adverse event occurred
9	Phase 3 (81)	Hepatitis A and/or B vaccine MenACWY-CRM	Germany	18–64 years, *N* = 252	• Concomitant administration of hepatitis A/B and MenACWY-CRM was non-inferior to administration of hepatitis A/B alone • hSBA titers ≥8 for serogroups A, C, W-135, and Y in the HepA/B+MenACWY-CRM group (76, 87, 99, and 94%, respectively) comparable to MenACWY-CRM group (67, 82, 96, and 88%, respectively) • No vaccine related serious adverse event reported
10	Phase 3 (82)	MenACWY-CRM	Korea	Adolescents, adults, *N* = 450	• Single dose of MenACWY-CRM showed hSBA titers ≥8 (79%, 99%, 98%, and 94% of subjects) and GMTs (48, 231, 147, and 107) against serogroups A, C, W, and Y, respectively, after 1 month
11	Phase 3 (83)	MenACWY-CRM	India	2–75 years, *N* = 180	• hSBA titers ≥8 were 72, 95, 94, and 90% and seroresponse rates 72, 88, 55, and 71% for serogroups A, C, W, and Y, respectively • GMTs increased 7-fold to 42-fold among the four serogroups.
**B. Vaccination in adults for Pneumococcal diseases**					
1	Phase 2 (84)	PCV20 PCV13/PPSV23	USA	60–64 years, *N* = 444	• Robust functional immune response for PCV20 group • OPA titers for all 20 serotypes included showed GMFRs from baseline (6.0 to 113.4) • AE and SAE rates were low and similar with PCV20 and PCV13 • In 1 month, OPA GMFRs for the 13 serotypes common to both PCV20 and PCV13 were lower for PCV20 compared with PCV13 (6.0–58.6 PCV20, 7.1–68.6 PCV13) • For seven serotypes unique to PCV20 and PPSV23, OPA GMFRs were higher for PCV20 (11.2–113.4 PCV20, 8.9–77.0 in the PPSV23) in 1 month
2	Phase 4 (85)	PCV13 QIV	USA	≥50 years, *N* = 882	• Non- inferior immune responses to all 13 pneumococcal serotypes and all four influenza strains with PCV13+QIV compared to each vaccine given alone, after 1 month • GMTs generally lower for PCV13+QIV compared to PCV13 alone
3	Phase 4 (86)	PPSV23 QIV	Not Specified	>65 years, *N* = 162 adults	• Simultaneous injections of PPSV23 and QIV (77.8%) or sequential PPSV23 injected 2 weeks after QIV vaccination (77.6%), showed non-inferior response rate • No significant decrease in seroprotection odds ratios for H1N1, H3N2, or B/Phuket influenza strains other than B/Texas, for simultaneous administration • No increase in systemic events and local reactions
4	Phase 3 (87)	V114 or PCV13, followed by PPSV23	USA, Korea, Spain, Taiwan	≥50 years, *N* = 652	• Comparable immune responses for V114 and PCV13 at 30 days post-vaccination with PPSV23 (Month 13) • Serotype-specific OPA higher in the V114 group than in the PCV13 group for the two unique V114 serotypes, 22F and 33F • For the 13 shared serotypes, OPA GMTs varied, but was comparable between V114 and PCV13 at Day 30 and at 12 months
5	Phase 3 (88)	PCV13 followed by PPSV23	USA	≥50 years, *N* = 400	• Both 2-month and 6-month dosing intervals of PCV13-PPSV23 well-tolerated • Serotype-specific OPA GMTs comparable in both dosing groups for six serotypes unique to PPSV23 and 12 serotypes shared between PCV13 and PPSV23 • No negative impact on immune responses after administration of PPSV23 following PCV13, regardless of interval • 2 months interval associated with higher frequency of injection-site AEs, after PPSV23, but most AEs were transient and mild to moderate in intensity
6	Phase 3 (89)	V114 QIV	USA	≥50 years, *N* = 1,200	• V114 administered concomitantly with QIV was non-inferior to V114 administered non-concomitantly with QIV, comparable OPA GMTs at 30 days post-vaccination with V114 for all 15 serotypes in V114 and all four influenza strains in QIV • Lower OPA GMTs in the concomitant group compared with the non-concomitant group observed
7	Phase 3 (90)	PCV13	Mexico	≥50 years, *N* = 324	• OPA GMTs increased significantly in 1 month after vaccination with PCV13 • The OPA GMFRs were higher in the 50–64-year age group (5.3–63.6) than those in the >65-year age group (3.4–35.8) • Pre-vaccination OPA GMTs in the 50–64-year age group were statistically significantly lower than >65-year age group for 8 of 13 serotypes, and similar for the five other serotypes • Post-vaccination OPA GMTs similar in both age groups for 10 of 13 serotypes, but statistically significantly higher for serotypes 4, 7F, and 9V in age group 50-64 years • Adverse reactions were significantly more frequent among those in the 50–64-years group, but mild to moderate in intensity
8	Phase 3 (91)	PPV23	China	>2 years, *N* = 1,760	A 2-fold increase rate of anti-pneumococcal antibody was observed for all 23 serotypes (range, 49.71–90.96%) in the test vaccine compared to control commercial vaccine (range, 44.52–88.24%)
9	Phase 3 (92)	PPV23	Russia	≥50 years, *N* = 100	Serotype-specific IgG mean concentrations increase 2-fold in ≥79.2%subjects (≥50 years of age) and ≥76.9 subjects (2–49 years of age) after single dose of PPV23
10	Phase 4 (93)	PCV13	India	50–65 years, *N* = 1,200	• Robust immune responses against all 13 pneumococcal serotypes with GMFRs (range, 6.6–102.7) of functional antibody GMTs before to 1 month after vaccination • No statistically significant differences in immune responses between age groups (50–59 years and 60-65 years)
11	Phase 3 (94)	PCV13	Japan	≥50 years, *N* = 271	• OPA assay titer GMFRs ranged from 11.5 (serotype 3) to 137.2 (serotype 7F) • GMFRs ranged from 7.3 to 99.9 and 13.6 to 192.5 for the 50–64 y and ≥65-year groups, respectively • Lower OPA GMTs in 50–64-year group than in the ≥65 years group for all serotypes, and statistically significantly lower for 8 of 13 serotypes
12	Phase 3 (95)	PCV13 PPSV23	Not specified	≥50 years, *N* = 831	• OPA GMT in the PCV13 group, post 1 month was statistically significantly higher than in the PPSV23 group for eight of the 12 serotypes common to both vaccines and for serotype 6A, unique to PCV13 • For the other four common serotypes the OPA GMT was comparable • Immune response to PCV13 was generally greater in 50–59 years of age compared to 60–64 years of age
13	Phase 4 (96)	PCV13	Not specified	≥65 years, *N* = 84,496	• PCV13 effective in prevention of vaccine-type community-acquired pneumonia and vaccine-type invasive pneumococcal disease • Vaccine efficacy was 46, 45, and 75% for the prevention of vaccine-type community-acquired pneumonia, non-bacteremic and non-invasive vaccine-type community-acquired pneumonia, and vaccine-type invasive pneumococcal disease, respectively
14	Phase 4 (97)	Td PCV13	Korea	≥50 years, *N* = 448	• Concomitant administration Td+PCV13 non-inferior in terms of GMT ratios for tetanus, diphtheria, and all four pneumococcal serotypes (1, 5, 18C, and 19A) • Higher IgG anti-tetanus antibody titer in Td alone group than Td+PCV13 group (50.7 vs. 26.5%) • OPA GMT comparable for PCV13 + Td and PCV13 alone groups, except for pneumococcal serotype 1, significantly higher in PCV13 + Td group
**C. Vaccination in adults of hepatitis B**					
1	Phase 3 (12)	3A-HBV and 1A-HBV	USA, Canada, Belgium, Finland, Germany, UK	18–45 years, *N* = 2,838	• SPR of 3A-HBV non-inferior to 1A-HBV • Higher SPR of 3A-HBV than 1A-HBV after 2 (90.4 vs.51.6%) and three vaccinations (99.3 vs.95%) • GMC of anti-HBs with 3A-HBV higher than 1A-HBV after 2 (7.9 times) and three vaccinations (3.5 times)
2	Phase 3 (98)	HBsAg-1018 or HBsAg-Eng	USA	18–70 years, *N* = 8,374	• Two doses of HBsAg-1018 demonstrated (total 4 weeks) higher (95.2– vs. 80.7%) SPR than three doses of HBsAg (in 24 weeks) • SPR was higher in the HBsAg-1018 group compared to HBsAg-Eng group (90.0 vs. 65%) in patients with type 2 diabetes
3	Phase 3 (99)	HBV-ISS HBV-Eng	Not specified	18–55 years, *N* = 2,415	• Two-dose regimen of HBV-ISS induced superior antibody response than a three-dose regimen of HBV-Eng • Higher percentage of subjects exhibiting a seroprotective immune response for HBV-ISS than for HBV-Eng at all time points (95.1 vs. 81.1%) • GMC of antibodies significantly higher in the HBV-ISS group
4	Phase 3 (100)	mpHBV, RECOMBIVAX-HB™ or ENGERIX-B™	Not specified	≥50 years, *N* = 540	• Group ≥65 years had lower vaccination responses than the 50–64-year group • SPRs were 82.1%, for mpHBV, 77.4% for RECOMBIVAX-HB™, and 88.5% for ENGERIX-B™, in 50–64 years group • SPRs were 57.5% for mpHBV, 34.4% for RECOMBIVAX-HB™, and 67.7% for ENGERIX-B™, in ≥65 years group
5	Phase 3 (101)	Heptavax^®^-II mpHBV	Japan	20–35 years, *N* = 722	• SPR of mpHBV SC non-inferior to Heptavax_- II SC • SPRs in the mpHBV SC group and Heptavax_- II SC group were 91.6 and 82.6%, respectively • anti-HBs GMTs higher in mpHBV SC compared to Heptavax_-II SC (231.4 and 91.2 mIU/mL, respectively) • SPR and GMTs in the mpHBV IM group was higher than both SC vaccine groups, at month 7 (SPR 98.7%, GMT 1,064 mIU/mL)
6	Phase 3 (102)	Nasvac Peg-IFN	Bangladesh	18–60 years, *N* = 160	• Higher ALT increase in Nasvac-treated than Peg-IFN treated subjects (85 vs. 30%), after 5 nasal vaccinations in subjects with chronic hepatitis B • Superior reduction of the viral load under the limit of detection, by Nasvac treatment compared to Peg-IFN treatment • Lower progression to cirrhosis in Nasvac group compared to Peg-IFN group • Nasvac significantly safer compared to Peg-IFN in all major variables related to AE
**D. Vaccination for adults in HPV**					
1	Phase 3b (103)	HPV-16/18 AS04-adjuvanted vaccine	Senegal, Tanzania	10–25 years, *N* = 676	• At month 7, 100% of initially seronegative participants were seropositive for both anti-HPV-16 and anti-HPV-18 antibodies • Seropositivity maintained till month 12 • GMTs for HPV-16 and HPV-18 were higher in 10–14-year-olds than 15–25-year-olds
2	Phase 3 (104)	qHPV	Colombia, France, Germany, Philippines, Spain, Thailand, and USA	24–45 years, *N* = 3,819	• Antibody titers against HPV 6, 11, 16, and 18 similar in women aged 24–34 years and 35–45 years • Overall, in 24–45-year group, 98% anti-HPV 6 seropositive, 98% anti-HPV 11 seropositive, 99% anti-HPV 16 seropositive, and 97% anti-HPV 18 seropositive, at month 7
3	Phase 3 (105)	qHPV	Colombia, France, Germany, Philippines, Spain, Thailand, and the United States	24–45 years, *N* = 3,819	• Vaccine efficacy against combined incidence of persistent infection, CIN/EGL related to HPV6/11/16/18 was 88.7% • At month 48, 91.5, 92.0, 97.4, and 47.9% of vaccinated women were seropositive to HPV 6/11/16/18, respectively
4	Phase 3 (106)	qHPV	Colombia	24–45 years, *N* = 1,360	• No cases of HPV 6/11/16/18-related CIN or EGL during the extended follow-up phase (post 6 years) • Month 72 total IgG seropositivity for HPV 6, 11, 16, and 18 was 87.8, 84.4, 98.8, and 81.5%, respectively
5	Phase 3 (107)	qHPV	Philippines or Thailand	24–45 years, *N* = 44 Mother-infant duo	• For each of HPV types 6, 11, 16, and 18, maternal anti-HPV was found in cord blood samples, which were highly positively correlated with maternal HPV titers • When mothers were seropositive at the time of birth, 84, 65, 100, and 62% of infants were seropositive for HPV-6,−11,−16, and−18, respectively
6	Phase 3 (108)	(HPV)-16/18 AS04-adjuvanted vaccine and qHPV	USA	18–45 years, *N* = 1,106 women	• For HPV-16, 100% subjects in HPV-16/18 vaccine group and majority (95.7–97.5%) in HPV-6/11/16/18 vaccine group were seropositive, at month 60 • For HPV-18, majority (98.1–100%) of subjects in HPV-16/18 vaccine group were seropositive, but seropositivity rates in HPV-6/11/ 16/18 vaccine group decreased considerably (61.1-76.9%) across the three age strata • Geometric mean titers for anti-HPV-16 and anti-HPV-18 antibodies were higher in HPV-16/18 vaccine group than in HPV-6/11/16/18 vaccine group
7	Phase 3 (109)	HPV-16/18 AS04-adjuvanted vaccine	USA, Canada, Belgium, France, Brazil, Australia, Finland, Germany, Italy, Mexico, Philippines, Spain, Taiwan, Thailand, UK	15–25 years, *N* = Around 18,000 women	• Following an initial peak at month 7, HPV-16, and HPV-18 GMTs were sustained throughout 36 months of follow-up • Negligible efficacy was observed for women aged 21–25 years, likely due to prevalent infections at baseline • HPV-16 and HPV-18 antibody titers post-vaccination tended to be higher among 15- to 17-year-olds than among 18- to 25-year-olds
8	Phase 3 (110)	qHPV	Australia, Austria, Brazil, Canada, Colombia, Czech Republic, Denmark, Finland, Germany, Hong Kong, Iceland, Italy, Mexico, New Zealand, Norway, Peru, Poland, Puerto Rico, Russia, Singapore, Sweden, Thailand, the UK, USA	15–26 years, *N* = 17,622 women	• Vaccination 100% effective in reducing the risk of HPV16/18-related high-grade cervical, vulvar, and vaginal lesions and of HPV6/11-related genital warts, in seronegative subjects • In the intention-to-treat group, vaccination significantly reduced the risk of any high-grade cervical lesions (19.0% reduction), vulvar and vaginal lesions (50.7%), genital warts (62.0%), Pap abnormalities (11.3%), and cervical definitive therapy (23.0%)
9	Phase 4 (111)	AS04-HPV-16/18 qHPV	Brazil, Estonia, India, Thailand	15–25 years, *N* = HIV females-873	• AS04-HPV-16/18 immunologically superior to qHPV in women living with HIV • Neutralizing anti-HPV-16 and HPV-18 antibody GMTs were 2.74 and 7.44-fold higher in AS04-HPV-16/18 than qHPV
10	Phase 2/3 (112)	AS04-HPV-16/18 vaccine	China	18–25 years, *N* = 6,051	• Initially seronegative women receiving HPV vaccine remained seropositive for anti-HPV-16/18 antibodies till month 72 • Long-term efficacy vaccine against infection, abnormal cytology and CIN lesions associated with HPV-16/18 up to 72 months after first vaccination
11	Phase 3 (113)	HPV 16/18 vaccine	USA, Canada, Australia, Mexico, Philippines, Thailand, UK, Netherlands, Peru, Portugal, Russia, Singapore	>26 years, *N* =5,752	• Significant vaccine efficacy against 6-month persistent infection or CIN1+ associated with HPV 16/18 in all age groups combined (90.5%), at month 84 • Cross-protective efficacy against 6-month persistent infection with HPV 31 (65.8%) and HPV 45 (70.7%) • 95% of initially seronegative women remained seropositive for HPV 16 and HPV 18, at month 84
12	Phase 3 (114)	HPV-16/18 AS04-adjuvanted vaccine	Germany, Poland	15–55 years, *N* = 488	• All women were seropositive for anti-HPV−16 and ≥97% for anti-HPV−18 antibodies, at 6 years after dose 1 • GMTs were higher (anti-HPV−16, 9.3–45.1-fold; anti-HPV−18, 4.3–19.4-fold) than natural infection levels, in all age groups
13	Phase 3 (115)	qHPV	China	20–45 years, *N* = 3,006	• Vaccine efficacy against HPV16/18-related CIN Grade 2 or 3, adenocarcinoma *in situ*, and cervical cancer (CIN 2+) and HPV6/11/16/18-related CIN 1+ was 100%, at month 78 • Efficacy against cervical 6- and 12-month persistent infection was 91.6 and 97.5% at Month 30 and Month 78, respectively • Rate of cervical cytology abnormalities associated with HPV6/11/16/18 reduced with an efficacy of 94.0%
14	Phase 3 (116)	9-valent HPV	Not specified	16–26 years, *N* = 2,520	• GMTs for HPV types 6, 11, 16, 18, 31, 33, 45, 52, and 58 for heterosexual men were non-inferior to those of women at month 7 • GMTs were lower in men having sex with men, than heterosexual men, for all vaccine HPV types, at month 7 • Over 99.5% of subjects were seropositive at Month 7 for each vaccine HPV type
15	Phase 3 (117)	qHPV	Not specified	15–45 years, *N* = 20,551 women	• No significant differences noted overall for the proportions of pregnancies resulting in live birth, fetal loss, or spontaneous abortion between vaccinated and placebo group • No negative affects with HPV6/11/16/18 vaccine in pregnancy outcomes
16	Phase 3 (118)	qHPV	Japan	16–26 years, 1,124 men	• At month 7, >97% of vaccinated participants seroconverted to each of the vaccine HPV types • Most participants remained seropositive at month 36, although the seropositivity rate declined between months 7 • Vaccine demonstrated 83.3 and 85.9% efficacy against HPV6/11/16/18-related persistent infection in the interim and final analyses, respectively

*ACWY, serogroups A, C, W-135 and Y; CV, conjugate vaccine; DT, diphtheria toxoid; GMT, geometric mean titer; hSBA, serum bactericidal activity (human complement); Men, meningococcal; PS, polysaccharide; rSBA, serum bactericidal activity (rabbit complement); TT, tetanus toxoid; VR, vaccine response; AE, adverse events; GMFRs, geometric mean fold rises; IgG, immunoglobulin G; OPA, opsonophagocytic activity; PCV, pneumococcal conjugate vaccine; PPV, pneumococcal polysaccharide vaccine; QIV, quadrivalent influenza vaccine; SAE, serious adverse events; ALT, alanine aminotransferases; GMC, geometric mean concentration; HBsAg, hepatitis B surface antigen; HBV, hepatitis B vaccine; mp, modified; 1A, 1antigen; 3A, 3antigen; Peg-IFN, pegylated interferon; SPR, seroprotection rate; CIN, cervical intraepithelial neoplasia; EGL, external genital lesions; qHPV, quadrivalent human papilloma virus; HIV, human immunodeficiency virus*.

### Meningococcal Vaccines

*N. meningitidis*, is associated with many clinical consequences such as meningitis, septicemia, and serious sequelae affecting life physically and psychologically (119). Colonization of the nasopharynx, called meningococcal carriage is common and leads to transmission and development of IMD. Carriage rates are found to be highest in adolescents and young adults (120, 121). Three types of vaccinations are available for IMD; polysaccharide, conjugate, and protein polysaccharide conjugate vaccines (monovalent, bivalent or multivalent). The vaccines are conjugated to carrier proteins such as tetanus toxoid, diphtheria toxoid, or diphtheria toxoid variant CRM197), which enhance immunological memory (18).

Vaccination programs have been highly successful at reducing the burden of serogroup C IMD in many countries, worldwide (122–124). In 1999, in response to the increasing number of cases of serogroup C IMD in the United Kingdom (UK), meningococcal C vaccine was added to the routine infant immunization schedule, increasing in demand for routine vaccination (125). Evidence from published literature suggests that mass vaccination campaigns using conjugated meningococcal vaccines around the world have led to control of serogroup C and a decrease in serogroup B disease (122). The authors noted that data from 10 years of surveillance (in countries with surveillance systems) following the introduction of the quadrivalent (A, C, W, Y) vaccine have led to control of serogroup C IMD in the UK, Canada, Australia, Spain, Belgium, Ireland, and Iceland. Since the introduction of the quadrivalent (A, C, W, Y) vaccine in the UK, there has been a 97% decrease in the incidence of meningococcal serogroup C IMD among both vaccinated and unvaccinated (herd effect) individuals (122). In the UK, there are epidemiologic data that suggest that the use of conjugate meningococcal C vaccines along with mass vaccination and catch-up programs have led to the protection of unvaccinated persons through herd immunity (126). Additionally, serogroup B IMD in New Zealand has also decreased, following the introduction of the serogroup B vaccine (122).

The introduction of mandatory quadrivalent ACWY vaccine in 2002 led to a significant decrease in number of IMD cases in Saudi Arabia. Between 1995 and 1999, the predominant serogroup was A, followed by B. However, between the 2000 and 2001 outbreaks of serogroup W-135 predominated, accounting for 78% of typed isolates. In the post-epidemic period, serogroups A and W were almost equally distributed (36 and 40%, respectively) (127). In England, the introduction of MenACWY vaccination program took place in 2015 and led to a decline of MenW as well as MenY cases' number in the post-immunization period (128). In Netherlands, the incidence of MenW has been reduced after the implementation of MenACWY in the immunization program. In Australia, meningococcal tetanus toxoid conjugate vaccine (MenACWY-TT) has been included into the National Immunization Program (NIP) in 2018, leading to a decline in overall meningococcal disease cases (129).

Quadrivalent polysaccharide vaccine, MenACWY has been conjugated with different carrier proteins, and have shown to be effective for adults in various studies, such as MenACYW-TT and meningococcal Diphtheria Toxoid Conjugate Vaccine (MenACWY-D) in adults aged 11–55 years (77). Other studies confirmed effectiveness of vaccines in adults >56 years (73, 76). In a study in Lebanon, conjugated vaccine (MenACWY-TT) and a quadrivalent polysaccharide vaccine (MensPS), were compared in terms of serum bactericidal activity (rabbit complement source; rSBA) vaccine response (VR) rates after 1 month. High VRs were observed for each serogroup after vaccination (93.3% and 100% in the MenACWY-TT group and MenPS group for serogroup A; 96.3 and 90.9% for serogroup C; 88.7 and 86.4% for W-135, respectively; and for Y, 100% in both groups) (73). The VR tends to decrease with age, as it was lower in subjects >65 years of age as compared to 56–65 years age group. The long-term effectiveness of conjugate vaccines was confirmed with persistence of immune response even after 5 years (74). Another quadrivalent polysaccharide–CRM197 conjugate vaccine (MenACWY-CRM), is currently approved for licensure in 65 countries in all age groups (82, 83, 130). A study in adults (19–65 years) in Latin America, compared MenACWY-CRM with MenACWY-D, and unconjugated meningococcal polysaccharide vaccine (MPSV4). The seroresponse rates were consistently higher in the MenACWY-CRM group than in the MenACWY-D group for all four serogroups, in the 19–55 years age group. Immune response was higher in the MenACWY-CRM group than in the MPSV4 group for serogroups A, C, and Y, in the subjects aged 56–65 years (130). Studies also support co-administration of vaccines, in adolescents and adults, which lowers healthcare visits, provides better adherence by young adults, and helps in accelerating pre-travel multi-vaccination schedules when traveling to endemic areas. MenACWY-CRM has been co-administered with tetanus diphtheria pertussis vaccine (Tdap), hepatitis A and/or B vaccine, and rabies vaccine and shown no safety concerns or interference with immune responses. Immunogenic responses to diphtheria, tetanus, and meningococcal (serogroups A, C, W-135, and Y) antigens were similar regardless of concomitant vaccine administration (81, 131). The conjugate vaccine (Men ACWY-TT) has also been co-administered with seasonal influenza vaccine in adults aged 18–55 years which induced robust immune responses, with at least 76.5% of subjects having a VR to each of the meningococcal serogroups, and a seroconversion rate of at least 61.9% to all three influenza strains (75).

Due to homology of MenB capsular polysaccharides to human neural cells, polysaccharide conjugate vaccines for serogroup B cannot be developed. Alternatively, two meningococcal serogroup B protein vaccines (MenB-4C; MenB-FHbp) can be administered as a 2-dose series (119). Lipoprotein 2086 which is a human complement factor H binding protein (fHBP), is used as a vaccine target for individuals 10–25 years of age. Immunogenicity of the vaccines was evaluated using human serum bactericidal assays (hSBA). Bivalent vaccine showed robust hSBA activity against diverse invasive meningococcus serogroup B disease strains and was well-tolerated (78, 132). Seroconversion rates from 70.0 to 94.7%, following the 3-dose vaccination, confirmed increase with subsequent vaccine doses (133). Coadministration of bivalent rLP2086 with diphtheria, tetanus, and acellular pertussis and inactivated poliovirus vaccine (DTaP/IPV) was immunologically non-inferior to DTaP/IPV administered alone. The vaccine showed robust and broad bactericidal antibody responses to diverse serogroup B strains after 2 and 3 vaccinations in adolescents and young adults (134).

### Pneumococcal Vaccines

*S. pneumoniae* is one of the most common causal pathogens of otitis media, sinusitis, conjunctivitis, and CAP in addition to causing more severe IPDs such as meningitis and sepsis. Other, less frequent infections caused by *S. pneumoniae* include periorbital cellulitis, osteomyelitis, endocarditis, pericarditis, peritonitis, pyogenic arthritis, soft tissue infections, and neonatal septicemia (53). A large proportion of IPD is vaccine preventable. Currently, two kinds of pneumococcal vaccines are available; namely, pneumococcal polysaccharide vaccines (PPVs) and pneumococcal conjugate vaccines (PCVs). Polyvalent polysaccharide vaccines were first developed and licensed based on the demonstrated efficacy of 6-valent and 13-valent vaccines against pneumococcal pneumonia in adults. Subsequently, both 14-valent and 23-valent vaccines were licensed based on comparable safety and immunogenicity profiles to lower valency vaccines for shared serotypes (88). Both types of vaccines are effective, but PPVs have a short-lived immune response. PCVs which have carrier protein conjugated to polysaccharide, induce B-cell memory response, and provide long-term immunity. 23-valent PPV (PPSV23) showed robust immune response in adults >50 years and individuals 2–49 years having high risk or with chronic conditions (91, 92). Efficacy of 13-valent PCV (PCV13) was observed among adults >65 years of age, in prevention of vaccine-type CAP and vaccine-type IPD which persisted till 4 years (96). Similarly, robust immune response was observed in other studies with single dose of PCV13 in adults >50 years of age (90, 93, 94). Jackson et al. showed PCV13 opsonophagocytic activity (OPA) titers (used to measure antibody response of the vaccine) were non-inferior to PPSV23 for all 12 common serotypes and significantly higher in eight serotypes for PCV13, in 60–65 years age group. PCV13 elicited a substantially greater response to serotype 6A, which is contained only in PCV13. High level of immune response and longer antibody persistence was observed with PCV13 in adults 50–59 years as compared to 60–65 years age group. Enhanced immunogenicity with PCV13 as compared to PPSV23 in adults confirms PCV13 has the potential for improved clinical efficacy against pneumococcal disease (95).

Although PCVs have significantly decreased pneumococcal disease worldwide; expanding serotype coverage may further reduce disease burden. A 15-valent vaccine, PCV15, was found to be non-inferior to PCV13 for 13 common serotypes and superior to PCV13 for two of its unique serotypes (22F and 33F) in adults >50 years of age (135, 136). A 20-valent PCV (PCV20) was developed to expand protection against pneumococcal disease beyond PCV13. PCV20 includes seven additional serotypes than PCV13, therefore providing broader coverage along with all advantages of conjugate vaccine. A study reported robust immune OPA response for PCV20 against all 20 serotypes in adults aged 60–65 years, with no serious adverse events, similar to PCV13 (84). Another Phase 3 study showed comparable OPA titers for all 20 serotypes and non-inferiority with PCV13, in adults aged 14–49 years, thus confirming the efficacy of PCV20 (137).

In adults aged >50 years, sequential dosing with PCV13 and PPSV23 or PCV15 with PPSV23, at 12-month intervals showed comparable immunogenic response, except for two unique serogroups of PCV15 (87, 88). Another study confirmed higher OPA titers with sequential dosing of PCV13 followed by PPSV23 (PCV13/PPSV23) than those following only an initial PPSV23; and with PPSV23 followed by PCV13 (PPSV23/PCV13) (138).

Simultaneous administration of vaccines has also been tested and is a cost-effective and promising strategy. For example, co-administration of PPSV23 with influenza vaccine (86), tetanus diphtheria (Td) with PCV13 (97), influenza vaccine with PCV13 (85) and influenza vaccine with PCV15 (89) have shown to be safe, well-tolerated, and without affecting immunogenicity as compared to separate administrations. This co-administration can have a greater protective effect in the elderly and high-risk groups and prevent hospitalization, morbidity, and mortality from severe respiratory diseases (86). PLHIV are at higher risk of pneumococcal infection and often require hospitalization. A systemic review stated immunization schedule consisting of a sequential combination of PCV13/PPSV23 [PCV13, followed by PPSV23 after minimum 8 weeks recommended by Advisory Committee on Immunization Practices (ACIP)] (139) in PLHIV, or having immunocompromising conditions, aged 19 years or older (140, 141). Also, for optimum vaccination response, vaccination with PCV should be delayed until immunological recovery (CD4 > 200/mm^3^) (140).

### *Haemophilus influenzae* Vaccines

*H. influenzae* is both a commensal and invasive pathogen of the upper respiratory tract, oropharynx, and nasopharynx. Major infections/diseases caused by *H. influenzae* include CAP, acute sinusitis, acute exacerbation of chronic bronchitis, and invasive infections such as bacteremia and meningitis (142, 143). These clinical manifestations are mostly caused by *H. influenzae* type b (Hib) strain, and/or non-typeable *H. influenzae* (NTHi) strain (144, 145). Vaccines against the Hib strain was developed as early as 1985 and has evolved over years with better efficacy, better immunogenicity, varied formulation choices and combinations (146). The combination vaccines protect against Hib disease, diphtheria, tetanus, pertussis (whooping cough), and polio, with additional hepatitis B vaccine in one of the combinations. Vaccination of Hib are either made of polysaccharides, the polyribosyl ribitol phosphate (PRP) of the Hib capsule which is less immunogenic in infants or conjugated as PRP linked to carrier proteins such as diphtheria toxoid or tetanus toxoid, making it more efficacious and immunogenic (147).

Hib vaccinations are recommended only for children younger than 5 years, and adults with certain medical conditions such as sickle cell anemia, HIV infection, asplenia, and cancer. Adult population is generally not given Hib vaccine and rely on natural boosting of immune response. The widespread use of Hib conjugate vaccines has drastically reduced invasive Hib disease, however the non-Hib diseases majorly caused by NTHi strain have increased. Invasive *H. influenzae* disease is now affecting adults, especially elderly and immunocompromised patients, more than children (148, 149). Hence, it is critical to develop and study vaccines against NTHi strain.

Acute exacerbation in patients with chronic obstructive pulmonary disease (COPD) is often associated with NTHi, therefore an investigational NTHi protein vaccine was tested in adults aged 40–80 years with moderate or severe COPD and a history of exacerbation. This study reported higher reactogenicity 7-day post-vaccination as compared to placebo, good immunogenicity, and acceptable safety profile. Further clinical assessments are required to evaluate effects on COPD symptoms and long-term outcomes (150). An early Phase I study was conducted to evaluate the safety and immunogenicity of a combined vaccine, NTHi-*Moraxella catarrhalis* (MCat) in adults with smoking history ≥10 pack-years, to immunologically represent the COPD population. The investigational NTHi-Mcat vaccine had an acceptable reactogenicity and safety with good immunogenicity in older adults with a smoking history. Further studies are warranted to assess the effectiveness of NTHi-Mcat vaccine (151). These potential vaccines against NTHi and other combination vaccines (*S. pneumoniae* and *H. influenzae* triple-protein vaccine) (152) may help to address the increasing burden of NTHi associated diseases.

### Hepatitis B Vaccines

The HBV infections lead to complications such as hepatocellular necrosis, cirrhosis, hepatocellular carcinoma, and chronic liver disease. Adults at risk of HBV infection including PLHIV, healthcare workers, and travelers to endemic countries are recommended for vaccination by CDC. Full vaccine coverage is essential to prevent infections, as only 35% of subjects who received one or two doses had protective antibody levels as compared to 65% in subjects who had received full three doses (153).

The first HBV vaccine developed in the early 1980's was plasma-derived which demonstrated effectiveness against HBV infection. However, unproven safety concerns regarding transmission of blood-borne pathogens (including HIV) hindered the acceptance of this vaccine in many populations (154). The recombinant second-generation HBV vaccine was developed to address this concern and to help produce large quantity of vaccines. The recombinant HBV vaccine produced from genetically engineered yeast contains the hepatitis B surface antigen (HBsAg), but lacks the preS domain and glycosylation present in the natural viral particle (155). This vaccine demonstrated excellent immunogenicity in all age groups from neonates to adults with a seroprotection rates (SPR) of 85–100% observed approximately after 1-month of final dose (2-dose schedule for adults) (156). However, SPR was lower in older adults, elderly, smokers, obese individuals, and patients with impaired immune function such as those with malignancies or undergoing hemodialysis (156). A more effective mammalian cell-derived vaccine contains the glycosylated pre-S1 and pre-S2 proteins, in addition to the major HBsAg protein. Currently, new adjuvant formulations have been and are being developed to increase the immunogenicity as compared to the second-generation HBV vaccines.

A few clinical studies comparing effectiveness of different hepatitis B vaccines in adults have been highlighted below. A study comparing tri-antigen to single-antigen vaccine in adults aged 18–45 years revealed robust immune response in tri-antigen vaccine as compared to single-antigen vaccine which took prolonged time to achieve seroprotection (12). Available HBV vaccines show variable immune responses due to presence of different adjuvants. In a study among healthy adults of age 18–55 years, two doses of a vaccine using HBsAg adjuvanted with an immunostimulatory phosphorothioate oligodeoxyribonucleotide (HBV-ISS) compared to three doses of an alum-adjuvanted vaccine (HBV-Eng) demonstrated higher SPR for HBV-ISS (99). One of the clinical studies evaluated the comparable effectiveness of variable dose of different HBV vaccines in high risk population. This study revealed that two doses of HBsAg-1018, which binds to toll-like receptors has higher seroprotection than three doses of HBsAg-Eng in elderly and patients with diabetes, obese, or smokers. HBsAg-1018 was able to induce seroprotection earlier compared to HBsAg-Eng (157). Another study demonstrated that two doses of HBsAg-1018 induced a higher SPR (90.0%) in a month in patients with diabetes than three doses of HBsAg-Eng over 6 months (65.1%) (98). Even in patients with chronic kidney disease and hemodialysis, who have high risk of HBV, three doses of HBsAg-1018 induced significantly higher and more durable seroprotection than four double doses of HBsAg-Eng (158).

Additionally, specific HBV vaccines were also assessed in patients with chronic disease conditions, for example HB-AS02 (an adjuvanted HBV vaccine), was administered in patients with renal insufficiency and other immunocompromised patients with low response to conventional recombinant HBV vaccines (159). In dialysis patients, three doses of HB-AS02 induced rapid and effective protection than a four-dose course of HB-AS04, which is the first adjuvanted HBV vaccine licensed for patients with renal insufficiency, in Europe. Another vaccine formulation consisting of both HBsAg and hepatitis B core antigen (HBcAg) was compared with use of pegylated interferon in chronic hepatitis B and was safer, better tolerated, and showed lower progression to cirrhosis (102). One of the major co-infections observed with HBV is HIV (~30% of PLHIV in sub-Saharan Africa). A study evaluating the response of HBV vaccines in adults with HIV (age group 21–84 years), observed that 80% of patients showed VR and it did not co-relate with age, CD4+ cell count or viral (160). Given the high risk to HBV exposure, the hepatitis B vaccine is strongly advised in PLHIV. The availability and option of different HBV vaccines for healthy adults as well as high risk populations has enabled us to overcome HBV infection, provided necessary vaccination strategies are employed.

### Human Papillomavirus (HPV) Vaccine

HPV can cause multiple epithelial lesions and cancers (161). It can clinically manifest as cutaneous and anogenital warts with a possibility to progress into cancer depending on the HPV subtype. Genital warts are most often (90% of cases) caused by infections due to HPV types 6 and 11 (162). Majority of cancers including cervical, anal, penile, oropharyngeal, vulvar, and vaginal cancers are caused by oncogenic HPV 16, 18, 45, and 31. HPV infections are mostly transmitted sexually (162). Currently, there are three vaccines available: bivalent (against HPV types 16 and 18), quadrivalent (against HPV types 6, 11, 16, and 18), and 9-valent (against HPV types 6, 11, 16, 18, 31, 33, 45, 52, and 58). The 9-valent HPV (9vHPV) has a broader coverage and provides immunity against most of the HPV infections. Currently, 9vHPV is recommended in both males and females in the age group of 9–25 years and adults 27–45 years only, if at risk (163).

A high prevalence of HPV has been reported among women in sub-Saharan Africa (164). Hence a clinical study to evaluate the efficacy and safety of bivalent HPV vaccines (2vHPV) was conducted on African girls and young women aged 10–25 years. The subjects were administrated three doses of bivalent, HPV-16/18 AS04-adjuvanted vaccine (AS04HPV-16/18) and all of them developed anti-HPV-16 and anti-HPV-18 antibodies and remained seropositive for 7 months (103). Several other studies have demonstrated high efficacy of 2vHPV against HPV 16/18-associated precancer and a long-term follow-up study (>10 years) have revealed persistence of antibodies ensuring that invasive cervical cancer is preventable with the use of recommended vaccinations (109, 114, 165, 166).

Several studies have showed efficacy of quadrivalent vaccine (4vHPV) in young adults. The HPV6/11/16/18 vaccine was 95–100% effective in reducing HPV16/18-related high-grade vaginal, cervical, and vulvar lesions, and 97% effective in reducing HPV6/11-related genital warts in women 15–26 years (110, 167). The efficacy and immunogenicity of 4vHPV was also seen in women aged 24–45 years (105). Long-term protection was confirmed, even a decade after administration (106, 168). Studies have confirmed that (2vHPV) is well-tolerated in pregnant women and presence of maternal antibodies in infants born to vaccinated women, for each of HPV types 6, 11, 16, and 18 (107, 117), confirming role of vaccination in decreasing prevalence in unvaccinated individuals also. Similar to the 2vHPV and 4vHPV, clinical studies have also confirmed efficacy of 9vHPV vaccines. Studies conducted on women aged 16–26 years have indicated that 9vHPV vaccine aided in preventing infection, cytological abnormalities, and high-grade lesions with sustained vaccine efficacy for 6 years (169, 170). Broader coverage provided by 9vHPV can dramatically decrease genital diseases and cervical cancer cases, worldwide (171, 172).

Women living with HIV are at greater risk of developing HPV infection and cervical cancer. Hence, a comparative study (2vHPV vs. 4vHPV vaccines) was performed on women living with HIV, aged 15–25 years. The outcomes of this study pointed out that though both vaccines were immunogenic, adjuvanted 2vHPV (AS04HPV-16/18) was immunologically superior to 4vHPV (HPV-6/11/16/18 vaccine) with higher HPV-16/18 antibody response and higher seropositivity rates after 24 months (111). Similar results were observed in healthy women of age 18–45 years with higher antibody responses with 2vHPV vs. 4vHPV even in the long-term (108).

The clinical studies in adults and elderly for the above mentioned VPDs have confirmed importance of including vaccination for adults in immunization schedules, with an aim to prevent VPDs and reduce mortality.

## Guidelines and Recommendations on Adult Vaccination

The vaccination recommendations for adults are categorized as a) recommendations for the general population and b) recommendations for populations with specific risk factors. The risk factors include pre-existing diseases (e.g., chronic lung or heart disease, diabetes mellitus), immunocompromised populations, travelers, and populations exposed to diseases as occupational risk (5).

The WHO emphasizes on immunization for all age groups. In 2012, WHO made recommendations for annual influenza vaccination defining specific groups at risk of influenza disease. Risk groups include pregnant women, health workers, children aged 6–59 months, persons 65 years of age and older, and individuals with specific chronic medical conditions (173). Effective boosters are recommended throughout older ages, especially in individuals with chronic respiratory comorbidities, can be introduced to lower the disease burden and prevent transmission to more vulnerable populations. For pregnant women, immunization with tetanus toxoid containing vaccine is recommended along with seasonal influenza vaccine. For adolescents aged between 9 and 19 years, immunization with diphtheria booster, HPV, tetanus booster along with typhoid is recommended. For adults and adolescents (9–19 years) immunization against cholera, dengue, rabies, and seasonal influenza are recommended (174). In the United States, the ACIP has made recommendations which are approved by the CDC and Prevention (175) and has been presented in Table 3.

**Table 3:** CDC recommended adult immunization schedule by age group, United States, 2021.

**Age**	**Recommended vaccines**
19–26 years	• Influenza activated (IIV) or Influenza recombinant (RIV4) or Influenza live (attenuated; LAIV4) → one dose annually • Tetanus, diphtheria, pertussis (Tdap or Td) → 1 dose Tdap each pregnancy; 1 dose Td/Tdap for wound management; 1 dose Tdap, then Td or Tdap booster every 10 years • Measles, mumps, rubella (MMR) → 1 or 2 doses depending on indication (if born 1957 or later) • Varicella (VAR) → 2 doses (if born in 1980 or later) • Human papilloma virus (HPV) → 2 or 3 doses depending on age at initial vaccination or condition • Pneumococcal conjugate (PCV13) 1 dose • Pneumococcal polysaccharide (PPSV23) → 1 or 2 doses depending on indication • Hepatitis A (Hep A) → 2 or 3 doses depending on vaccine • Hepatitis B (Hep B) → 2 or 3 doses depending on vaccine • Meningococcal A, C, W, Y (MenACWY) → 1 or 2 doses depending on indication • Meningococcal B (MenB) → 2 or 3 doses depending on vaccine and indication • *Haemophilus influenzae* type b (Hib) → 1 or 3 doses depending on indication
27–49 years	• Influenza activated (IIV) or Influenza recombinant (RIV4) or Influenza live (attenuated; LAIV4) → one dose annually • Tetanus, diphtheria, pertussis (Tdap or Td) → 1 dose Tdap each pregnancy; 1 dose Td/Tdap for wound management; 1 dose Tdap, then Td or Tdap booster every 10 years • Measles, mumps, rubella (MMR) → 1 or 2 doses depending on indication (if born 1957 or later) • Varicella (VAR) → 2 doses (if born in 1980 or later) • Human papilloma virus (HPV) → 2 or 3 doses depending on age at initial vaccination or condition (for age group 27 years to 45 years) • Pneumococcal conjugate (PCV13) → 1 dose • Pneumococcal polysaccharide (PPSV23) → 1 or 2 doses depending on indication • Hepatitis A (Hep A) → 2 or 3 doses depending on vaccine • Hepatitis B (Hep B) → 2 or 3 doses depending on vaccine • Meningococcal A, C, W, Y (MenACWY) → 1 or 2 doses depending on indication • Meningococcal B (MenB) → 2 or 3 doses depending on vaccine and indication • *Haemophilus influenzae* type b (Hib) → 1 or 3 doses depending on indication
50–64 years	• Influenza activated (IIV) or Influenza recombinant (RIV4) → one dose annually • Tetanus, diphtheria, pertussis (Tdap or Td) → 1 dose Tdap each pregnancy; 1 dose Td/Tdap for wound management; 1 dose Tdap, then Td or Tdap booster every 10 years • Measles, mumps, rubella (MMR) → 1 or 2 doses depending on indication (if born 1957 or later) • Varicella (VAR) → 2 doses • Zoster recombinant (RZV) → 2 doses • Pneumococcal conjugate (PCV13) → 1 dose • Pneumococcal polysaccharide (PPSV23) → 1 or 2 doses depending on indication • Hepatitis A (Hep A) → 2 or 3 doses depending on vaccine • Hepatitis B (Hep B) → 2 or 3 doses depending on vaccine • Meningococcal A, C, W, Y (MenACWY) → 1 or 2 doses depending on indication • Meningococcal B (MenB) → 2 or 3 doses depending on vaccine and indication
	• *Haemophilus influenzae* type b (Hib) → 1 or 3 doses depending on indication
≥65 years	• Influenza activated (IIV) or Influenza recombinant (RIV4) → one dose annually • Tetanus, diphtheria, pertussis (Tdap or Td) → 1 dose Tdap each pregnancy; 1 dose Td/Tdap for wound management; 1 dose Tdap, then Td or Tdap booster every 10 years • Varicella (VAR) 2 doses • Zoster recombinant (RZV) → 2 doses • Pneumococcal conjugate (PCV13) → 1 dose • Pneumococcal polysaccharide (PPSV23) → 1 dose • Hepatitis A (Hep A) → 2 or 3 doses depending on vaccine • Hepatitis B (Hep B) → 2 or 3 doses depending on vaccine • Meningococcal A, C, W, Y (MenACWY) → 1 or 2 doses depending on indication • Meningococcal B (MenB) → 2 or 3 doses depending on vaccine and indication • *Haemophilus influenzae* type b (Hib) → 1 or 3 doses depending on indication

The CDC has also provided recommendations for various immunization practices based on medical conditions. All the vaccines mentioned in Table 3 are recommended for asplenia (complement deficiencies), end-stage renal disease or patients on hemodialysis, patients with heart or lung disease, alcoholic patients, patients with chronic liver disease, diabetic individuals, healthcare personnel, and men engaging in sex with men. Influenza live attenuated vaccine (LAIV4) is not recommended in pregnancy, immunocompromised patients (excluding HIV) and in PLHIV. Measles, mumps, rubella (MMR) and varicella (VAR) are not recommended in pregnant women and immunocompromised patients (excluding HIV), and for PLHIV with a CD4 count of <200 mm^3^, while HPV is contraindicated in pregnant women. Pregnant women are encouraged to get immunization with Influenza inactivated vaccine (IIV) or Influenza recombinant (RIV4), Tetanus, diphtheria, pertussis (Tdap) or Td, PPSV23, Hepatitis A, HBV, and MenACWY vaccines. Caution is to be exercised for vaccinating pregnant women with MenB. Immunocompromised patients who do not have HIV infection are advised to get vaccinated with IIV or RIV4, Tdap or Td, HPV, PCV13, PPSV23, HepA, HepB, MenACWY, MenB, and Hib. PLHIV can get immunized with IIV or RIV4, HPV, PCV13, PPSV23, HepA, HepB, MenACWy, MenB, and Hib. Additionally, PLHIV with CD4 count of ≥200 mm^3^ can get vaccinated by MMR and VAR (175).

There are substantial differences country-wise in terms of vaccination schedules for adult population, which depend on many factors such as economic policies and overall incidence of disease including prevalent serotypes in the region. As of 2019, 17 European countries have implemented obligatory vaccination policies for adults. In most of the countries, vaccination policies for influenza, tetanus, diphtheria, pneumococcal disease, hepatitis B, and pertussis are present. Several European countries recommend MenACWY and MenB meningococcal vaccination for adolescents, as MenB is most common cause of IMD in age group 15–25 years (176). The vaccination schedule for the European Union countries can be found at: https://vaccine-schedule.ecdc.europa.eu/. In some countries, such as Australia and New Zealand, meningococcal vaccination is recommended for adolescents and adults only in special conditions, such as residing in accommodations as hostels or in military or having certain medical conditions or traveling to endemic countries. Recommendations and guidelines are also revised every few years based on the clinical requirements and available evidence (119). For instance, recommendations by ACIP, 2014 mandate the use of PCV13 in series with PPSV23 for all adults aged ≥65 years (177). However, this recommendation was revised in 2019, due to the reduced disease burden. According to the updated recommendations, routine administration of PCV13 in adults >65 years are removed and may be administered based on mutual decision considering patient's risk for exposure and underlying medical conditions (178). Additionally, ACIP recommends PCV13 in series with PPSV23 for adults ≥19 years having immunocompromizing conditions, cerebrospinal fluid leak, or cochlear implant (≥1 year between pneumococcal vaccines) (178). Recently, the ACIP updated their recommendations for age-based and risk-based use of pneumococcal vaccines among adults (139) and voted for the following recommendations: (i) Adults aged ≥65 years who have not previously received a PCV or whose previous vaccination history is unknown should receive a PCV (either PCV20 or PCV15). If PCV15 is used, this should be followed by a dose of PPSV23, (ii) Adults aged 19–64 years with certain underlying medical conditions or other risk factors who have not previously received a PCV or whose previous vaccination history is unknown should receive a PCV (either PCV20 or PCV15). If PCV15 is used, this should be followed by a dose of PPSV23 (139).

## Overview of Vaccination Practices in Tunisia and Morocco

There is a lack of adult vaccination practices in LMIC of Africa and Asia, but with the emerging trends in adult vaccination globally, there is a paradigm shift. Many countries are adopting vaccination practices from established high-income countries and adapting it based on the regional requirements. Global Alliance for Vaccines and Immunization (GAVI) is bringing together public and private sectors with the shared goal of saving lives and protecting people's health by increasing equitable and sustainable use of vaccines. GAVI supports low-income countries to help introduce vaccines into their NIP. In Tunisia and Morocco, the vaccination drive is usually conducted through the NIP. However, the NIPs are more focused on pediatric vaccination, while the adult vaccination programs are limited.

### Meningococcal Vaccines

According to the WHO global summary on VPDs monitoring system in 2019, the Moroccan immunization program does not include meningococcal vaccines (179). However, response plan exists for vaccination in the event of a case of meningitidis or in the event of an epidemic. Also, systematic prevention by the anti-meningococcal vaccine is applied as part of the national control program to protect people living in institutions and pilgrims (180). For people living in institutions, high risk closed communities such as prisons, orphanages, charitable homes, boarding schools, and possibly other closed communities depending on the level of promiscuity, meningococcal vaccines are recommended for all people who have not been vaccinated or who have been vaccinated before 3 years without any age restriction (180). For pilgrims, MenACWY is highly recommended. Moroccan pilgrims are obliged to present a certificate of vaccination against meningitis with a quadrivalent ACW135Y vaccine, received at least 10 days before departure (181). Bivalent polysaccharide vaccine against serogroups A and C, conjugate vaccine against serogroup C, and tetravalent vaccine against serogroups A, C, W, and Y are available. However, no vaccine is available for serogroup B (180).

Similarly in Tunisia, meningococcal vaccines are not included in the NIP (179). Polysaccharide conjugate MenACWY vaccine is the only available meningococcal vaccine in Tunisia and is recommended for Hajj pilgrims and travelers to endemic areas and to children considered at high risk (17, 18).

### Pneumococcal Vaccines

Morocco and Tunisia have introduced PCVs in their NIP (182, 183). In Morocco, the 13-valent PCV was introduced in October 2010 and replaced by the 10-valent PCV in July 2012 in the NIP (183). But the NIP does not include adult pneumococcal vaccination.

### Hemophilus Influenza Vaccines

Hib vaccine has been introduced in Morocco through its routine immunization program in 2007 for children aged <5 years. Then, in 2012 pentavalent DTC-Hib-HB was introduced including diphtheria, tetanus, pertussis, Hib, and hepatitis B (184, 185).

In Tunisia, Hib vaccine was reintroduced in 2011 as Hib conjugate in combination with hepatitis B, diphtheria, poliomyelitis, and tetanus called the pentavalent vaccine, under the NIP (186).

### HPV Vaccines

The HPV vaccine was licensed in Morocco in 2008, but it is not affordable by most and not yet included in NIP. Moreover, its awareness and acceptance among adolescents and adults is very low (22). The HPV vaccine is recommended for young girls aged 11–14 years, with a booster dose after 5 years and ideally completed before the start of sexual activity (187).

The HPV vaccine is not included in NIP of Tunisia in spite of high number of cases in adults (23).

### Hepatitis Vaccines

Hepatitis B vaccine is included in Moroccan NIP for children. For adults, it is strongly recommended for healthcare workers, but significant number is still not vaccinated (188).

In Tunisia, as a part of the NIP, hepatitis B vaccination is provided for healthcare workers (189).

### Tetanus, Diphtheria, Pertussis, and Poliomyelitis Vaccines

In Morocco, NIP includes national tetanus vaccination schedule for elderly women 15–45 years old. For adolescents and women of childbearing age who have already received a primary vaccination and boosters, according to the NIP (five doses), a 6th dose is recommended to ensure lifelong protection. For adolescents and women of childbearing age who have never been vaccinated or whose information is uncertain, they should be vaccinated according to the national schedule recommended for this group (five doses) (185).

Tunisian NIP covers diphtheria, tetanus, and oral polio vaccine at the age of 18 years and vaccination for rubella and tetanus for women of childbearing age (15–45 years) (190). The Global Pertussis Initiatives have recommended immunization against pertussis for all healthcare workers in African countries (70).

### Influenza Vaccines

Morocco joined Partnership for Influenza Vaccine Introduction (PIVI) in 2014 and prioritized influenza vaccines for the elderly, healthcare professionals, healthcare students, and those with chronic illnesses such as diabetes in their 2014 vaccination campaign (191). The National Immunization Technical Advisory Group issued recommendation for the seasonal influenza vaccine and requested continuous burden data generation (21). Also, as overcrowding is considered as a factor favoring the transmission of respiratory pathologies, specifically flu, vaccination against this disease is strongly recommended for all pilgrims, especially the elderly and those with chronic illnesses (diabetics, respiratory failure, etc.) (181). Indeed, an annual dose of influenza vaccine is recommended for high-risk patients (21, 192). Still, it is estimated that only less than half of some risk groups may receive the influenza vaccine annually in Morocco, hence every prospect to promote the vaccination should be undertaken (193).

In Tunisia, influenza vaccine is recommended by Ministry of Health for elderly and healthcare workers, still its uptake is considerably low (192). Influenza vaccine is also recommended for pilgrims (192).

### Other Vaccinations

In Morocco, combined measles and rubella vaccine (RR) was introduced in schools in 2003. National campaigns for RR were organized occasionally for children aged 9 months-15 years and girls aged 15–24 years. From 2015, the NIP covers measles and RR for children <5 years (at 9 and 18 months) and includes catch-up vaccination, when needed, for the family of premature children (194).

Pasteur Institute of Morocco manages vaccinations including rabies vaccine and vaccines for travelers, such as yellow fever vaccine. It also provides individual or mass vaccination services through vaccination campaigns (periodically against influenza and throughout the year against viral hepatitis B, tetanus and meningitis) (195).

The Pasteur Institute of Tunisia manages vaccinations for travelers to endemic areas and at high risk. It includes vaccination for meningitis, diphtheria, tetanus, rabies, measles, mumps, tuberculosis, typhoid, hepatitis A, hepatitis B, polio, flu, and Yellow fever (196). There are also special recommendations for vaccination for workers in Tunisia, which includes mandatory immunization for typhoid, hepatitis B, and tetanus.

Despite the aforementioned vaccination guidelines and recommendations, there are still clinical challenges and gaps in the implementation and monitoring of these processes.

### Critical Clinical Gaps

Elderly population suffer from more serious consequences of VPDs, being highly sensitive to infections, making the treatment and vaccination programs clinically imperative. Further, antimicrobial resistance (AMR) which represents a global threat, is having increasing incidence. This can be attributed to inappropriate use of antimicrobials, lack of infection and disease control measures, poor access to medicines, vaccines and diagnostics, and lack of awareness (197). Vaccination is an important tool that will help reduce the spread of antimicrobial resistance (10).

The lack or insufficiency in clinical surveillance for adult's VPDs in African countries is another major unmet need. Around 75% of the reported meningococcal infections in the region lack confirmatory laboratory testing (198). To tackle these gaps, there is an immediate need for the development and reinforcement of surveillance systems to enable integration of public/private healthcare with respect to epidemiology, laboratory, and data management (15). This would support in developing enhanced vaccination strategies with improved outcomes.

There are also other factors greatly impacting the clinical applicability of vaccination in adults in the North African countries including gaps in recommendations from health professionals, poor information on the risks of the vaccine compared to the benefits of disease prevention, the gap in coordinated immunization programs for some VPDs in adults and missed opportunities during hospital visits, the absence of clinics or hospital departments for routine adult immunization and the weak basic training of physicians on adult immunization. Availability of certain vaccines at high cost or only in private sectors, also leads to low uptake.

## Economic Impact of Vaccine Preventable Diseases

The economic burden of VPDs related to 10 recommended vaccines among the United States adults, aged ≥19 years, was reported to be ~$9 billion in 2015, and about $7.1 billion (80%) was accrued by unvaccinated individuals (199). Another study on the economic impact of VPDs such as influenza, pertussis, herpes zoster, and pneumococcal disease in populations aged >50 years has been projected to increase from ~$35 billion to $49 billion over the next three decades, in the United States (200). Costs associated with VPDs depend on various factors such as geographic region and may be categorized as direct, indirect, and societal. Estimated average costs per IMD case during outbreaks from 1990 to 2010 in high-income and low-income countries were $41,857 to $55,755 and $2,222, respectively. In sub-Saharan Africa, the average cost per household per IMD case was estimated at $90-$244 depending on significant sequelae costs (16). Most of the studies on the economic burden of VPDs and cost-effectiveness of vaccines for pneumococcal infection and influenza have been limited to high-income countries and have demonstrated cost saving and clinical superiority (201, 202). In one of the few studies estimating cost-effectiveness of PCV vaccination in Tunisia, both PCV10 and PCV13 are expected to generate $1.8 million and $2.24 million respectively, in direct medical costs savings, compared to no vaccination (183). Economic evidence supports cost-effectiveness of PCVs in LMIC, including Tunisia (183, 203, 204). Although, the cost-effectiveness of adult vaccination has been established globally, the major challenges are still the lack of awareness and the cost of the vaccines in LMIC. High cost of vaccines, such as Tdap and PCV13 in Tunisia and HPV in Morocco, makes implementation logistically difficult. A major challenge faced by countries not eligible for assistance from GAVI is providing all recommended vaccines for adults through NIP (15). There is an imperative need to support the development of national guidelines and implement effective vaccination policies in LMIC to overcome these challenges.

Additionally, the paucity of data related to health economic assessments of adult vaccination programs triggers the need to develop techniques to access direct and indirect costs of VPDs that significantly affect the adult population. The complex adult vaccination schedule along with limited vaccination infrastructure often act as a barrier. Another challenge was associated with the determination of vaccine coverage and screening coverage causing an economic burden (205).

## Conclusion

Infectious diseases are still the leading cause of death in Africa and Asia. Lack of effective vaccination program among adults poses a huge challenge to control and prevent VPDs. Some of the major factors involved in low vaccination coverage among adults include high cost of vaccines, lack of awareness, fear of side effects, lack of management for developing new vaccines, handling, storage, and distribution of vaccines. In Morocco and Tunisia, lack of surveillance techniques, and screening facilities/laboratories are additional clinical gaps that needs to be addressed to boost vaccination. There is also a need of cost-effectiveness studies in Tunisia and Morocco to advocate adult immunization as these can be important analysis tools to understand clinical and economic impact of vaccination which will further emphasize the benefits of vaccination programs, strengthen reliability, and retain public trust.

Implementation of vaccination for adults and elderly would be highly beneficial as it helps decrease mortality, reduce infections, and prevent cancers such as cervical cancer or hepatocellular carcinoma. Initiatives on spreading awareness and clinical benefits of adult vaccination by both government and non-governmental agencies, may help improve adherence to scheduled and routine immunization programs. Overall, effective governance on local, national, and international levels, together with evidence-based development of policies may help to minimize the challenges of adult vaccination and improve coverage.

## Author Contributions

JR and HZ contributed to medical writing. RA, MB, MC, and KM have substantially contributed to the development of the manuscript by providing critical insights. All authors helped with conceptualization, data curation, review, editing of the manuscript, and read and approved the final manuscript.

## Funding

This work including article processing fee for the journal was sponsored by Pfizer Inc.

## Conflict of Interest

JR was employed by Pfizer Inc., Casablanca, Morocco. HZ was employed by Pfizer Inc., Tunis, Tunisia. The remaining authors declare that the research was conducted in the absence of any commercial or financial relationships that could be construed as a potential conflict of interest.

## Publisher's Note

All claims expressed in this article are solely those of the authors and do not necessarily represent those of their affiliated organizations, or those of the publisher, the editors and the reviewers. Any product that may be evaluated in this article, or claim that may be made by its manufacturer, is not guaranteed or endorsed by the publisher.
